# Test–Retest Reliability of Task Performance for Golf Swings of Medium- to High-Handicap Players

**DOI:** 10.3390/s22239069

**Published:** 2022-11-23

**Authors:** Israel Villarrasa-Sapiña, Nuria Ortega-Benavent, Gonzalo Monfort-Torres, Jesús Ramon-Llin, Xavier García-Massó

**Affiliations:** 1 Human Movement Analysis Group (HuMAG), Universidad de Valencia, 46010 Valencia, Spain; 2 Departamento de Educación Física y Deportiva, Universidad de Valencia, 46010 Valencia, Spain; 3 Departamento de Educación Física, Artística y Música, Universidad de Valencia, 46010 Valencia, Spain; 4 Unidad de Educación, Florida Universitaria, 46470 Catarroja, Spain

**Keywords:** biomechanics, reliability, measurement, methodology, performance

## Abstract

Background: Golf swing performance in medium- to high-handicap players must be reliably measured to use this variable in both research studies and in applied settings. Nevertheless, there are no studies published on this topic and test–retest evidence is only available for low-handicap players. The aim of this study was to determine the number of attempts necessary to obtain a reliable measurement protocol for swing performance variables in medium- to high-handicap players. Methods: Ten amateur players (55.67 (13.64) years, 78.4 (11.4) kg, 1.75 (7.95) m) took part in a test–retest study in two experimental sessions one week apart. In each one, fifteen swings with a six iron and a driver were evaluated with a 3D Doppler tracking golf radar. Results: The results showed that variables related to side carry could not be reliably measured in medium- to high-handicap players in only fifteen trials (ICC < 0.26, SEM > 12.05 m and MDC > 33.41 m). The rest of the performance variables related to the club and ball trajectories could be reliably measured with a 3D Doppler radar with between seven and ten swings. Conclusions: At least seven swings are recommended for the driver and ten for the six iron to measure golf swing performance.

## 1. Introduction

Golf has become increasingly popular among amateur players [1]. At the beginning of the 21st century, there were about 30,000 golf courses and 55 million players around the world [2], while in 2021, this number had increased to 66 million [1]. This popularity has given rise to scientific studies aiming to determine the most common injuries in golf players and the most important biomechanical and physiological determinants of swing performance. The swing used depends on the type of shot played. There are three main swing shots: full swings, approach or chip shots and putt shots [3].

Swing performance is usually measured using variables related to club and ball trajectories, as well as their interaction. These variables may include the club head speed, total distance, carry distance, spin loft and smash factor. Readers who want a deeper description of swing performance variables can read [4]. Several studies determining the effects of different training protocols on swing performance have been published [5,6,7,8,9,10,11,12,13,14]. One of the key methodological issues that should be addressed to obtain robust conclusions is the reliability of the protocol used to measure full swing performance, but very few of the published studies have provided a specific reliability analysis for the testing protocols used [5,6,7,8,9,10].

Most of these studies measured different performance variables in between three- to five-trial protocols and analyzed their reliability using the interclass correlation coefficient (ICC). Read et al. measured club head speed in a three-trial protocol with low-handicap players (i.e., 5.8 (2.26)) and obtained good reliability results (i.e., ICC = 0.87) [10], while other studies used a five-trial protocol with these players (i.e., ≤5) and obtained excellent ICC values (i.e., ICC > 0.9) [5,6,7,8]. Weston et al. used a ten-trial protocol to quantify club head speed, backspin and sidespin with participants with a medium handicap (i.e., 11.2 (6.1)), which provided moderate to good reproducibility indices (ICC = 0.68–0.84) [9]. It should be noted that the studies on medium-handicap players obtained less reliable results with ten-trial protocols than the studies on low-handicap players, even with fewer trials.

There are other non-experimental studies that have quantified swing parameters and analyzed their reliability [15,16,17,18,19]. Schofield et al. measured downswing velocity with a three-trial protocol and obtained good to excellent ICC values (i.e., 0.7–0.98), depending on the load [15]. Lewis et al. used a three-trial protocols to study the “maximal” tee shots in low-handicap participants and obtained good to excellent ICC results (i.e., ICC = 0.85–0.95) [16]. Gordon et al. studied club head speed in low-handicap players (i.e., ≤8) and obtained excellent ICC values (i.e., ICC = 0.95) with a five-shot protocol [18], while Barnett et al. [19] developed a process-oriented test to assess golf swing and putt stroke in children and obtained acceptable test–retest reliability (i.e., ICC = 0.6).

Moreover, only one study provided reliability data for an assessment protocol of five shots considering a wide variety of performance variables (i.e., club head speed, face angle, club path, attack angle, ball speed, carry, side carry and spin rate). In this study with low- to medium-handicap (i.e., 9.3 ± 8) players, Outram and Wheat found that, using five good shots with a driver, five iron and nine iron, the ICC was between 0.64 and 0.97 for all the performance variables [20]. It should be noted that the reliability data for ball and club performance variables during swing were obtained with low- to medium-handicap players and it is possible that higher handicap players would require a different number of strokes to obtain reliable results. This is because the variability in the swing performance of low-handicap players is lower than that of high-handicap players [21], and variability is a key issue for reliability.

The players’ level is not the only factor than can influence test–retest reliability. The number of swings completed in the measurement protocol is another key factor. There are several studies (not only on golf swing) that have analyzed the effect of the number of trials performed in the experimental protocol on the test–retest reliability [22,23]. With this analysis, reliability studies can provide recommendations on the number of trials required to carry out a reliable measurement.

As far as the authors are aware, only one study has examined the effect of the number of shots used to compute biomechanical variables related to golf performance on reliability [20,22]. Severin et al. performed a reliability study that established the number of trials required to achieve good reliability based on the ICC, standard error of the measurement and sequential averaging analysis in low- to medium-handicap players (i.e., 7.8 ± 4.7) using a six iron and a driver [22]. The results showed that the number of trials required needed to measure range of motion, angular velocity, ground reaction forces and torques during the golf swing varied from 4 to 11. It should be noted that these are biomechanical variables but not performance variables per se.

Thus, there is a gap in the literature with respect to the number of trials required to obtain reliable performance measurements with medium- to high-golf-handicap players. This information can be crucial for both researchers and coaches who need to evaluate swing performance in this population.

There is thus a need for reproducibility studies that provide sufficient information to establish the best golf swing measurement protocols to measure swing performance variables for participants with high handicaps. The contribution of this study is the determination of the test–retest reliability of swing performance variables for medium- to high-handicap players. Moreover, the number of swings needed to evaluate the performance of golf swings in this population with good reliability was determined. The aim of this study was thus to determine the number of attempts necessary to obtain a reliable measurement protocol for swing performance variables with medium- to high-handicap players. We hypothesized that more trials would be needed to obtain reliability than found previously with low- to medium-handicap players.

## 2. Materials and Methods

### 2.1. Participants

Sample size was determined using the equation proposed by Bonett et al. [24] and the previously published ICC value (i.e., 0.86) for seven-iron and driver club speeds and distances [5]. The significance level was set at *p* = 0.05 at 80% statistical power, which meant ten subjects participated in the study. Recruitment was undertaken with a non-probabilistic method (convenience sample). Eight subjects were male and two female, with a mean age of 55.67 (13.64) years, weight of 78.4 (11.4) kg, height of 1.75 (7.95) m and body mass index of 25.62 (2.44) kg/m^2^. The participants’ mean handicap was 31.23 (6.63) strokes. The inclusion criteria were: (i) players with a handicap between 18 and 36 strokes and (ii) players who did not have an injury during the 6 months prior to the study (self-reported).

All the participants voluntarily gave their consent to participate and the protocols applied in this research project were approved by our university’s Ethical Committee.

### 2.2. Tasks and Apparatus

The participants performed the tasks described below with a six iron and a driver in two experimental sessions separated by one week to allow the test–retest reliability to be determined between the two experimental sessions. The driver was selected because it is one of the golf clubs most frequently used both by practitioners of the sport and in studies [5,6,7,8]. The six iron was selected because it is an intermediate iron and is also used in studies that carry out this type of analysis [22]. At the beginning of the first session, the subjects were informed about the study on arrival and signed the informed consent form. They then undertook a warm-up (10 min) based on joint mobility and low-intensity muscle-resistance exercises, as well as taking practice swings with their own six iron and driver [10]. The experimental task consisted of performing 15 “good” shots with the maximum effort, first with the six iron and then the driver, aiming at an 80 × 80 cm target. “Good” shots were self-determined by the participants in accordance with their perception and assessment. Any trial that the participant reported to be a miss hit or considered unsatisfactory in any other way was excluded from the analysis [22]. The participants used their own clubs during the tests and were allowed 30–60 s of rest between trials. Once they had completed the 15 “good” shots with the six iron, they rested for 5 min and then started the driver shots. The self-perceived ”bad” trials were excluded from the analysis.

**Figure 1:**
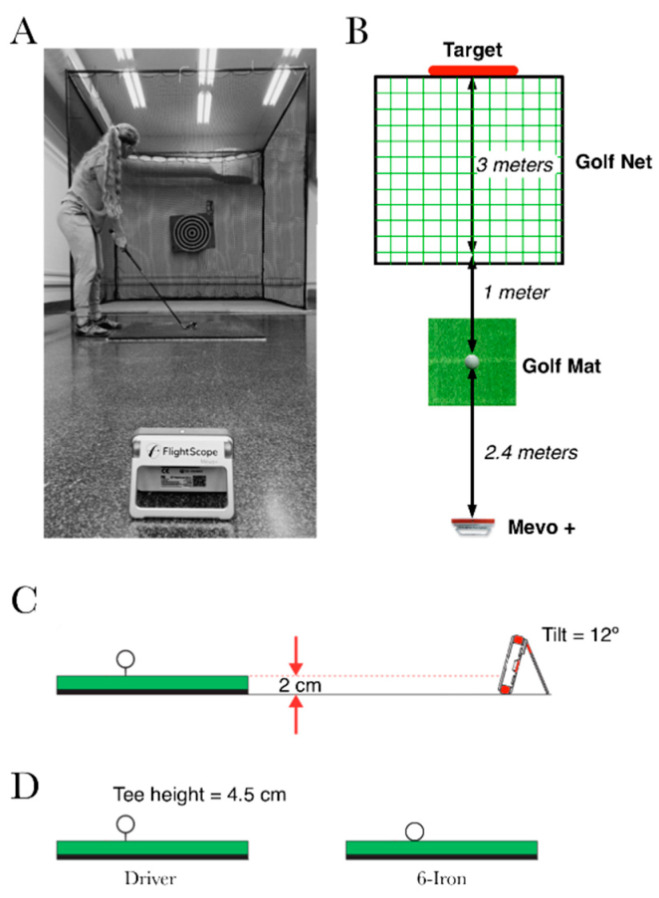
Experimental setting. (**A**) picture from the back of the experimental setting in the lab is shown in layer (**A**). Layer (**B**) presents a top view of the setting with the distances between the Mevo+, the golf mat, the golf net and the target. In layer (**C**), a lateral view of the Mevo+ is shown, as well as the height difference between the sensor and the golf mat and the tilt of the Mevo+. Finally, in layer (**D**), the positions of the ball on the golf mat during the driver and six iron shots are shown.

The laboratory was equipped with a golf cage (3 × 3 × 3 m) with a safety net to stop the golf ball after each shot. The participants were placed at 1 m from the cage wall and the ball was placed on an artificial grass mat (1 × 1 m). When the participants performed the driver shots, the ball was placed on a rubber tee. A 3D Doppler tracking golf radar (FlightScope Mevo+, FlightScope EDH, Orlando, FL, USA) was placed 2.4 m behind the middle of the golf mat (Figure 1), as recommended by the manufacturer for indoor measurements. An 80 × 80 cm target was placed behind the net.

The golf radar recorded the performance variables of the subjects’ shots, which were stored using the FS golf App (FlightScope EDH, Orlando, FL, USA). The performance parameters included were: ball speed, club speed, smash factor, carry distance, total distance, roll distance, spin rate, apex height, flight time, angle of attack, spin loft, spin axis, lateral distance, launch direction and launch angle [4]. The data parameters are explained on the manufacturer’s website (https://flightscopemevo.com/pages/flightscope-data-parameters (accessed on 8 November 2022)).

### 2.3. Statistical Analysis

The statistical analysis was carried out in Matlab R2021b (Mathworks, Natick, MA, USA). Three test–retest reliability parameters were computed: the intraclass correlation coefficient (A,k model [25]), the standard error of the measurement (SEM) and the minimum detectable change (MDC). These parameters were computed using the first trial from the test and retest days, as well as the mean value from 2 to 15 trials in the two testing sessions. This procedure has been performed previously to determine the number of shots needed to reach acceptable reliability values for swing biomechanical variables [22]. ICC scores lower than 0.5 were interpreted as poor, those between 0.5 and 0.75 as moderate and those between 0.75 and 0.9 as good, while scores greater than 0.9 were interpreted as excellent reliability [26]. To determine the number of trials required to compute each of the performance variables, a new index was created (see Equation (1)) called the required trials index (RTI) that could be used for this type of reliability study:

(1) $$RTI_{i}=\frac{\left(\frac{\left(ICC_{1}-ICC_{i}\right)}{ICC_{1}}-1\right)\cdot 100}{\left(\sqrt{i}+1\right)}$$

where *ICC* is the intraclass correlation coefficient, *i* is the number of trials used to compute the ICC and *ICC*1 is the ICC obtained with only one trial. The same index was obtained using the SEM and MDC values by simply changing the −1 of the numerator to +1. Then, the numbers of trials with the highest RTI values for the SEM and MDC and the lowest RTI value for the ICC were selected for each performance parameter. These differences were due to the fact that greater ICC values indicated greater reliability while, in the case of the SEM and MDC, the lower values indicated greater reliability. The mean values and standard deviations of all the performance variables were calculated using the required number of trials, which, according to the RTI, were seven for the driver and ten for the six iron. Student’s t-test was applied for related samples to compare the means of the test and the retest for each variable. The level of significance was set at *p* = 0.05.

## 3. Results

**Figure 2:**
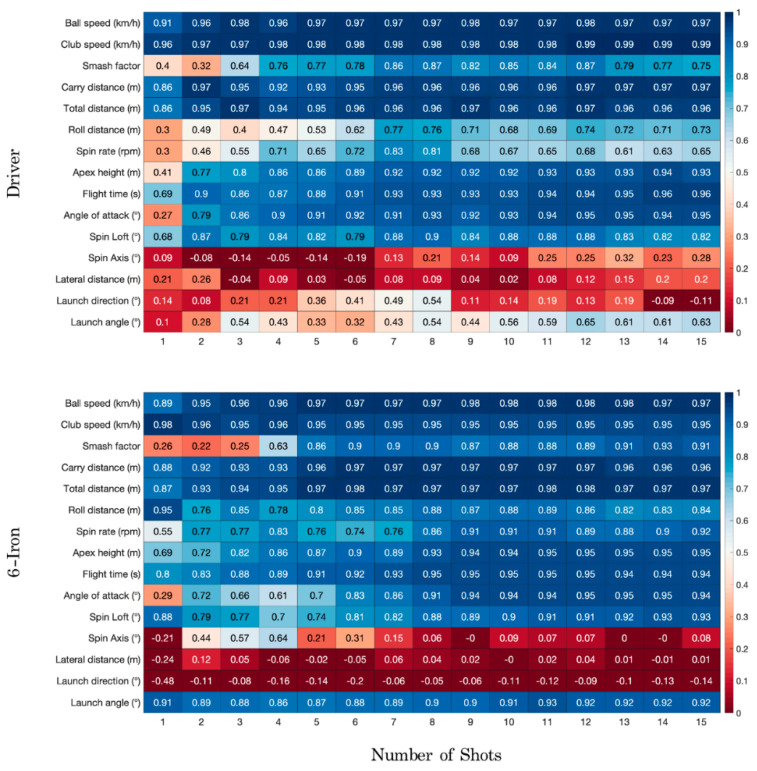
Heatmap representing the ICC values for each golf club and performance parameter using one to fifteen swings. Red colors indicate poor reliability values (below 0.5), while blue tones indicate moderate to excellent reliability values (above 0.5). The number included in each cell represents the ICC value for each performance parameter depending on the number of shots used to compute it.

Figure 2 shows the ICC values for the driver and six iron using 1 to 15 shots for each swing parameter. It can be seen that some parameters reached excellent reliability with only one or two shots (i.e., ball speed, club speed, carry distance and total distance), others needed between three and eight to reach good to excellent values (i.e., smash factor, roll distance, spin rate, apex height, flight time, angle of attack, spin loft) and others did not reach acceptable values (i.e., spin axis, lateral distance and launch direction). Furthermore, it seemed that some variables could be measured more reliably using six iron shots than with the driver (e.g., launch angle).

The SEM and MDC results are shown in Table 1 and Table 2 and indicate the same tendency as the ICC results. Some parameters with poor to moderate ICC values showed relatively low SEM values; for example, launch direction values varied from 1.2 to 5.55 degrees. This indicates that not only should relative reliability variables (i.e., ICC) be taken into account when test–retest reliability is discussed but also absolute ones (i.e., SEM and MDC).

It should be noted that the number of required trials (RTI) differed when ICC, SEM and MDC were used to compute it (Table 3). For example, if we check the values of Table 3 for the smash factor of driver swings, the RTI recommended four shots using ICC as the reliability variable and seven when the variable used was SEM or MDC. Considering all the data from Table 3 as a whole, seven trials can be recommended for a driver and ten for a six iron in order to reach good reliability values (i.e., ICC, SEM and MDC) for almost all variables. Only spin axis and launch direction with the driver needed more than seven trials. Nevertheless, these variables did not reach good reliability values independently of the number of trials chosen (see Figure 2 and Table 1 and Table 2). Therefore, seven trials can be recommended for protocols with the purpose of measuring drive performance. Regarding the six iron, spin loft, launch direction and launch angle needed 11, 12 and 11 trials, respectively. Nevertheless, the reliability values for spin loft and launch angle were also excellent with ten trials. Finally, launch direction did not reach good reliability values independently of the number of shots considered for its measurement. Overall, ten trials seems to be an adequate recommendation to measure swing performance with a six iron.

The mean and standard deviation of the parameters calculated using seven trials for the driver and ten trials for the six iron are shown in Table 4, as well as the results of Student’s t-test for samples related between the test and retest. As can be seen, there were no significant differences between the test and retest values for any variable.

## 4. Discussion

This is the first study to establish the protocol needed to reach test–retest reliability in measurements to assess performance parameters related to golf swing in medium- to high-handicap players, and the results obtained have important implications for research on how the performance of novice amateur players can be improved and how trainers can measure players’ performance.

First, good reliability could be achieved in all the performance parameters except for spin axis, lateral distance and launch direction, which probably need more than fifteen shots due to their great variability. In relation to this, Severin et al. performed a reliability study that established that the range of motion, angular velocity and ground reaction force variables (biomechanical variables related to swing performance) could be reliably measured in low- to medium-handicap players [22]. Outram and Wheat performed a reliability analysis of ball and club trajectory variables (similar to our variables) in low- to medium-handicap players and found good to excellent ICC values when including five swings in the analysis. In their study, lateral distance achieved good reliability (i.e., ICC = 0.7–0.73 and SEM = 1.7–2.4 m, depending on the golf club used), which contrasts with the poor values obtained in the present study (i.e., ICC < 0.26, SEM > 12.05 m and MDC > 33.41 m, regardless of the number of swings and the golf club). This difference was probably due to the fact that the variables related to lateral carry are the most difficult to control for novice players [21]. In this sense, Weston et al. found a lower ICC for spin axis (0.68) than spin loft or club head speed (i.e., 0.72–0.84), as well as a greater typical error (30.3%, 12.2% and 4.4% for spin axis, spin loft and club head speed, respectively) [9]. It should be noted that the sample in this previous study was composed of players with a medium handicap (i.e., 11.2), while in the present study, the players had a higher handicap (i.e., 31.23). It is therefore possible that lateral distance cannot be reliably measured in medium- to high-handicap players using a laboratory protocol and a Doppler radar system or that more than fifteen swing trials may be needed.

Secondly, for the rest of the performance variables, the number of required swings could be set at seven for the driver and ten for the six iron. It should be noted that these values were established using ICC, SEM and MDC values and not only ICC values. For example, although carry distance could be measured with a single swing for the six iron according to the ICC value (i.e., 0.88), the MDC varied from 31.53 m for one trial to 17.97 m for the mean value of six trials. This study recommends more trials than the five trials used by Outram and Wheat in their reliability study [20]. These differences could have been due to the disparity in the skill levels between the golfers in the two studies. In this sense, Read et al. found excellent reliability (i.e., ICC = 0.87) with low-handicap players when club head speed was measured with a driver using three trials [10]. Other studies using five trials also found excellent values for club head speed with low-handicap players (i.e., ICC > 0.9) [5,6,7,8]. Nevertheless, Weston et al. found excellent reliability for club head speed (i.e., ICC = 0.84) with medium-handicap players (i.e., 11.2) but they had to perform ten trials [9]. Therefore, these results suggest that the lower the handicap of the players, the lower the number of trials required to reach good reliability. This hypothesis was confirmed by our results, since seven to ten trials were required to reliably measure swing performance in high-handicap players. These numbers of trials were higher than those recommended for low-handicap players (i.e., three to five trials) [5,6,7,8,10,20] and similar to those found for medium-handicap players (i.e., ten trials) [9]. Finally, Severin et al. recommended between 4 and 12 trials for low-handicap players to measure kinematic and kinetic variables during swing performance [22]. Nevertheless, these biomechanical variables (performance variables) were not included in the present study, so no direct comparisons can be made.

Finally, we want to highlight the contribution of this study to establishing the method to be applied for test–retest studies that aim to determine the number of trials required in a motor task to reach reliable measurements [27]. The required trial index can be used to select the best number of trials to achieve reliable measurements while minimizing the measurement time (i.e., as few trials as possible). This index was proposed and used in this study to avoid subjective interpretations by the researchers. Future studies should test the performance of this index in other studies that examine the reliability of different motor actions and variables.

This study had some limitations that should be highlighted. As only fifteen trials were performed, it was not possible to obtain a reliable protocol for the parameters related to lateral deviation. It is possible that more reliable values could have been obtained for these variables with a greater number of shots.

## 5. Conclusions

It can be concluded that the performance variables related to the club and ball trajectories of medium- to high-handicap golf players performing between seven and ten swings could be reliably measured using a 3D Doppler radar. At least seven swings are recommended for the driver and ten for the six iron. The variables related to lateral deviation could not be reliably measured with medium- to high-handicap players using fifteen swings.

## Figures and Tables

**Table 1:** Standard error of the measurement for FlightScope Mevo+ extracted parameters.

		Number of Shots														
		1	2	3	4	5	6	7	8	9	10	11	12	13	14	15
Driver	Ball speed (km/h)	11.03	7.53	5.05	7.36	6.40	6.15	5.77	5.76	5.68	6.20	6.25	5.92	6.48	6.70	6.44
	Club speed (km/h)	5.00	4.40	4.10	3.75	3.48	3.31	3.09	3.20	3.08	3.05	3.13	2.87	2.70	2.68	2.78
	Smash factor	0.08	0.07	0.05	0.04	0.04	0.04	0.03	0.02	0.03	0.03	0.03	0.03	0.04	0.04	0.04
	Carry distance (m)	15.16	7.82	9.30	11.63	10.90	9.58	8.60	8.73	8.52	8.95	8.75	8.39	8.27	8.19	7.80
	Total distance (m)	15.54	9.08	7.36	9.79	9.31	8.59	7.79	7.78	7.64	8.02	7.99	7.79	8.28	8.37	8.08
	Roll distance (m)	7.77	5.37	6.19	5.83	5.14	4.17	3.32	3.32	3.53	3.72	3.58	3.26	3.25	3.35	3.21
	Spin rate (rpm)	1389.99	1012.47	814.27	626.94	622.91	524.44	386.68	392.22	497.05	488.77	494.48	484.19	536.63	513.98	506.72
	Apex height (m)	6.85	4.10	4.00	3.40	3.32	2.81	2.52	2.40	2.46	2.47	2.34	2.31	2.32	2.24	2.26
	Flight time (s)	0.64	0.38	0.48	0.47	0.45	0.38	0.34	0.34	0.34	0.35	0.33	0.31	0.29	0.28	0.27
	Angle of attack (°)	1.25	0.76	0.67	0.59	0.59	0.55	0.54	0.45	0.48	0.42	0.39	0.37	0.36	0.39	0.36
	Spin loft (°)	7.47	3.71	4.45	3.68	3.87	3.99	2.97	2.77	3.47	3.03	2.94	2.97	3.66	3.81	3.78
	Spin axis (°)	41.97	22.83	23.23	19.07	17.24	18.04	13.75	12.00	13.58	13.48	11.48	12.05	10.79	12.35	11.58
	Lateral distance (m)	22.26	12.26	17.51	12.05	14.87	15.89	15.51	15.18	17.25	16.55	14.66	15.38	14.15	13.24	12.94
	Launch direction (°)	3.65	2.36	2.13	1.79	1.46	1.39	1.34	1.20	1.76	1.66	1.47	1.73	1.77	2.20	2.20
	Launch angle (°)	7.58	4.62	3.13	3.66	3.60	3.32	3.02	2.75	2.95	2.72	2.55	2.34	2.49	2.50	2.46
Six Iron	Ball speed (km/h)	10.68	7.43	6.75	6.55	5.66	5.32	5.38	5.45	4.98	5.09	4.97	4.91	5.13	5.25	5.24
	Club speed (km/h)	3.81	4.80	5.29	4.85	4.99	4.98	5.09	5.17	5.25	5.20	5.10	5.13	5.06	4.91	4.88
	Smash factor	0.08	0.06	0.07	0.04	0.03	0.02	0.02	0.02	0.03	0.02	0.02	0.02	0.02	0.02	0.02
	Carry distance (m)	11.38	9.72	9.17	8.91	7.35	6.48	6.37	6.05	6.04	6.08	5.78	5.93	6.38	6.48	6.62
	Total distance (m)	11.42	8.27	7.73	7.31	5.79	5.05	5.21	5.30	5.09	5.15	4.94	4.93	5.19	5.31	5.44
	Roll distance (m)	0.47	1.89	1.77	2.30	2.01	1.75	1.84	1.52	1.65	1.58	1.44	1.67	1.91	1.85	1.77
	Spin rate (rpm)	541.00	360.32	405.48	329.12	404.22	446.13	438.74	334.92	282.22	285.20	276.17	309.92	324.10	284.83	259.84
	Apex height (m)	3.15	3.51	3.01	2.77	2.64	2.47	2.53	1.97	1.88	1.91	1.81	1.82	1.80	1.80	1.80
	Flight time (s)	0.43	0.47	0.42	0.40	0.37	0.35	0.33	0.27	0.27	0.28	0.26	0.28	0.29	0.29	0.29
	Angle of attack (°)	1.96	1.46	1.16	1.16	1.12	0.83	0.75	0.62	0.52	0.55	0.54	0.48	0.50	0.49	0.52
	Spin loft (°)	4.91	5.84	5.11	5.59	4.94	4.04	3.90	3.08	3.03	2.91	2.71	2.77	2.67	2.50	2.48
	Spin axis (°)	9.01	11.42	8.08	6.77	10.39	9.45	10.63	11.25	12.21	11.07	10.69	10.75	11.15	10.42	9.63
	Lateral distance (m)	12.82	16.29	15.91	19.80	19.34	16.62	16.77	16.79	16.70	15.42	15.20	14.12	15.55	15.59	14.47
	Launch direction (°)	4.20	4.70	4.88	5.55	4.82	4.49	4.43	4.32	4.15	3.88	3.93	3.81	4.14	4.35	4.12
	Launch angle (°)	1.86	1.90	1.94	2.05	1.78	1.63	1.61	1.45	1.50	1.41	1.21	1.32	1.31	1.34	1.33

**Table 2:** Minimum detectable change for FlightScope Mevo+ extracted parameters.

		Number of Shots														
		1	2	3	4	5	6	7	8	9	10	11	12	13	14	15
Driver	Ball speed (km/h)	30.57	20.87	14.01	20.41	17.73	17.05	16.00	15.97	15.74	17.19	17.32	16.41	17.97	18.59	17.84
	Club speed (km/h)	13.87	12.21	11.36	10.39	9.63	9.17	8.56	8.86	8.54	8.45	8.67	7.96	7.47	7.43	7.71
	Smash factor	0.23	0.20	0.14	0.11	0.10	0.10	0.07	0.07	0.08	0.08	0.08	0.08	0.11	0.11	0.11
	Carry distance (m)	42.02	21.67	25.79	32.25	30.22	26.55	23.85	24.19	23.61	24.79	24.24	23.26	22.93	22.70	21.61
	Total distance (m)	43.07	25.18	20.41	27.13	25.79	23.80	21.60	21.58	21.17	22.23	22.15	21.58	22.94	23.21	22.39
	Roll distance (m)	21.54	14.87	17.15	16.15	14.25	11.56	9.21	9.21	9.80	10.32	9.93	9.04	9.01	9.30	8.90
	Spin rate (rpm)	3852.85	2806.42	2257.05	1737.79	1726.61	1453.66	1071.81	1087.18	1377.74	1354.80	1370.63	1342.09	1487.47	1424.67	1404.55
	Apex height (m)	19.00	11.38	11.09	9.44	9.19	7.79	6.98	6.66	6.83	6.85	6.48	6.40	6.43	6.20	6.26
	Flight time (s)	1.77	1.06	1.32	1.32	1.25	1.05	0.94	0.94	0.95	0.98	0.92	0.87	0.81	0.78	0.75
	Angle of attack (°)	3.47	2.11	1.85	1.64	1.63	1.52	1.51	1.24	1.33	1.16	1.08	1.03	0.98	1.08	1.00
	Spin loft (°)	20.70	10.30	12.34	10.21	10.74	11.06	8.23	7.68	9.61	8.38	8.14	8.23	10.15	10.55	10.49
	Spin axis (°)	116.33	63.27	64.39	52.86	47.78	50.00	38.12	33.26	37.65	37.36	31.82	33.40	29.89	34.23	32.09
	Lateral distance (m)	61.71	33.99	48.54	33.41	41.22	44.06	43.00	42.06	47.83	45.88	40.64	42.64	39.23	36.69	35.86
	Launch direction (°)	10.13	6.54	5.91	4.95	4.04	3.86	3.73	3.31	4.87	4.59	4.09	4.79	4.91	6.10	6.09
	Launch angle (°)	21.01	12.79	8.68	10.16	9.97	9.19	8.38	7.62	8.17	7.55	7.07	6.50	6.91	6.93	6.81
Six Iron	Ball speed (km/h)	29.61	20.59	18.70	18.16	15.70	14.76	14.90	15.09	13.81	14.12	13.78	13.62	14.21	14.55	14.53
	Club speed (km/h)	10.56	13.31	14.66	13.43	13.82	13.80	14.10	14.32	14.55	14.41	14.13	14.23	14.03	13.60	13.53
	Smash factor	0.24	0.18	0.19	0.12	0.08	0.07	0.06	0.06	0.07	0.07	0.07	0.07	0.06	0.05	0.06
	Carry distance (m)	31.53	26.96	25.41	24.71	20.36	17.97	17.66	16.77	16.74	16.86	16.02	16.45	17.69	17.97	18.34
	Total distance (m)	31.65	22.93	21.43	20.26	16.04	14.01	14.43	14.69	14.10	14.27	13.69	13.65	14.38	14.72	15.09
	Roll distance (m)	1.31	5.23	4.91	6.38	5.58	4.84	5.09	4.21	4.58	4.38	4.00	4.64	5.31	5.13	4.91
	Spin rate (rpm)	1499.57	998.77	1123.95	912.27	1120.43	1236.62	1216.12	928.36	782.28	790.54	765.50	859.07	898.36	789.52	720.24
	Apex height (m)	8.72	9.72	8.35	7.68	7.32	6.84	7.02	5.45	5.21	5.29	5.01	5.05	4.99	4.98	5.00
	Flight time (s)	1.18	1.31	1.16	1.12	1.02	0.97	0.92	0.75	0.76	0.77	0.73	0.77	0.81	0.82	0.82
	Angle of attack (°)	5.42	4.05	3.20	3.23	3.09	2.29	2.09	1.71	1.44	1.52	1.50	1.33	1.39	1.35	1.43
	Spin loft (°)	13.62	16.18	14.16	15.50	13.69	11.21	10.81	8.54	8.39	8.05	7.52	7.68	7.41	6.94	6.88
	Spin axis (°)	24.99	31.66	22.40	18.76	28.79	26.20	29.46	31.18	33.86	30.69	29.63	29.81	30.92	28.89	26.69
	Lateral distance (m)	35.52	45.17	44.09	54.88	53.60	46.06	46.49	46.54	46.29	42.73	42.12	39.14	43.11	43.22	40.11
	Launch direction (°)	11.64	13.03	13.52	15.40	13.36	12.43	12.28	11.98	11.51	10.77	10.88	10.55	11.47	12.05	11.43
	Launch angle (°)	5.15	5.27	5.38	5.67	4.94	4.50	4.46	4.02	4.16	3.92	3.35	3.66	3.64	3.72	3.67

**Table 3:** Number of shots required for the calculation of each performance parameter depending on the reliability index used.

Club	Performance Parameter	ICC	SEM	MDC
Driver	Ball speed (km/h)	1	3	3
	Club speed (km/h)	1	7	7
	Smash factor	4	7	7
	Carry distance (m)	1	2	2
	Total distance (m)	1	3	3
	Roll distance (m)	7	7	7
	Spin rate (rpm)	4	7	7
	Apex height (m)	2	7	7
	Flight time (s)	2	2	2
	Angle of attack (°)	2	4	4
	Spin loft (°)	2	2	2
	Spin axis (°)	13	2	2
	Lateral distance (m)	1	2	2
	Launch direction (°)	8	5	5
	Launch angle (°)	3	3	3
Six Iron	Ball speed (km/h)	1	6	6
	Club speed (km/h)	1	1	1
	Smash factor	5	6	6
	Carry distance (m)	1	6	6
	Total distance (m)	1	6	6
	Roll distance (m)	1	1	1
	Spin rate (rpm)	2	2	2
	Apex height (m)	1	9	9
	Flight time (s)	1	8	8
	Angle of attack (°)	2	9	9
	Spin loft (°)	1	11	11
	Spin axis (°)	1	4	4
	Lateral distance (m)	1	1	1
	Launch direction (°)	1	12	12
	Launch angle (°)	1	11	11

ICC = intraclass correlation coefficient; SEM = standard error of the measurement; MDC = minimum detectable change.

**Table 4:** Mean, standard deviation and reliability values of the performance parameters using the required number of trials for the computation.

Club	Performance Parameter	Test	Retest	t9	*p*-Value	ICC	SEM	MDC
Driver (Seven Shots)	Ball speed (km/h)	175.77 (34.83)	178.39 (36.55)	−0.71	0.5	0.97	5.77	16
	Club speed (km/h)	132.91 (23.21)	131.17 (24.16)	0.88	0.4	0.98	3.09	8.56
	Smash factor	1.33 (0.08)	1.36 (0.06)	−1.64	0.14	0.86	0.03	0.07
	Carry distance (m)	129.60 (41.56)	134.16 (45.63)	−0.84	0.42	0.96	8.6	23.85
	Total distance (m)	140.52 (39.68)	143.90 (42.30)	−0.68	0.51	0.96	7.79	21.6
	Roll distance (m)	11.00 (6.09)	9.75 (7.79)	0.64	0.54	0.77	3.32	9.21
	Spin rate (rpm)	3803.54 (1008.35)	3978.11 (881.99)	−0.76	0.47	0.83	386.68	1071.81
	Apex height (m)	16.68 (8.99)	17.91 (8.35)	−0.79	0.45	0.92	2.52	6.98
	Flight time (s)	4.57 (1.29)	4.79 (1.34)	−1.05	0.32	0.93	0.34	0.94
	Angle of attack (°)	2.95 (1.67)	2.83 (1.98)	0.36	0.72	0.91	0.54	1.51
	Spin loft (°)	22.46 (8.75)	21.65 (8.35)	0.44	0.67	0.88	2.97	8.23
	Spin axis (°)	23.89 (11.64)	26.58 (17.78)	−0.41	0.69	0.13	13.75	38.12
	Lateral distance (m)	13.76 (6.47)	20.12 (25.84)	−0.77	0.46	0.08	15.51	43
	Launch direction (°)	4.57 (1.94)	4.88 (1.83)	−0.43	0.68	0.49	1.34	3.73
	Launch angle (°)	15.29 (4.43)	15.35 (3.61)	−0.04	0.97	0.43	3.02	8.38
Six Iron (Ten shots)	Ball speed (km/h)	144.68 (31.34)	140.34 (33.84)	1.43	0.19	0.98	5.09	17.19
	Club speed (km/h)	115.56 (21.43)	112.26 (23.53)	1.03	0.33	0.95	5.20	8.45
	Smash factor	1.25 (0.07)	1.24 (0.07)	0.42	0.69	0.88	0.02	0.08
	Carry distance (m)	100.58 (32.21)	94.52 (35.46)	1.75	0.12	0.97	6.08	24.79
	Total distance (m)	105.15 (30.29)	100.04 (32.81)	1.73	0.12	0.97	5.15	22.23
	Roll distance (m)	4.57 (3.61)	5.54 (5.53)	−1.01	0.34	0.88	1.58	10.32
	Spin rate (rpm)	5488.64 (898.82)	5476.29 (957.06)	0.07	0.95	0.91	285.20	1354.80
	Apex height (m)	15.55 (6.93)	14.60 (8.28)	0.80	0.45	0.94	1.91	6.85
	Flight time (s)	4.34 (1.10)	4.14 (1.28)	1.26	0.24	0.95	0.28	0.98
	Angle of attack (°)	6.33 (2.22)	6.77 (2.14)	−1.33	0.22	0.94	0.55	1.16
	Spin loft (°)	36.47 (8.83)	37.66 (9.55)	−0.66	0.53	0.90	2.91	8.38
	Spin axis (°)	12.21 (8.20)	16.12 (15.06)	−0.74	0.48	0.09	11.07	37.36
	Lateral distance (m)	7.53 (4.27)	17.45 (26.53)	−1.17	0.27	0.00	15.42	45.88
	Launch direction (°)	3.23 (1.25)	6.77 (6.11)	−1.74	0.12	−0.11	3.88	4.59
	Launch angle (°)	18.58 (4.09)	18.8 (5.26)	−0.26	0.80	0.91	1.41	7.55

ICC = intraclass correlation coefficient; SEM = standard error of the measurement; MDC = minimum detectable change.

## Data Availability

Not applicable.
